# PICK-ing Malaysia’s Epidemic Apart: Effectiveness of a Diverse COVID-19 Vaccine Portfolio

**DOI:** 10.3390/vaccines9121381

**Published:** 2021-11-24

**Authors:** Jing Lian Suah, Peter Seah Keng Tok, Su Miin Ong, Masliyana Husin, Boon Hwa Tng, Sheamini Sivasampu, Thevesh Thevananthan, Maheshwara Rao Appannan, Faizah Muhamad Zin, Shahanizan Mohd Zin, Hazlina Yahaya, Norhayati Rusli, Mohd Fikri Ujang, Hishamshah Mohd Ibrahim, Noor Hisham Abdullah, Kalaiarasu M. Peariasamy

**Affiliations:** 1 COVID-19 Immunisation Task Force, Government of Malaysia, Putrajaya 62000, Malaysia; boonhwa@bnm.gov.my (B.H.T.); thevesh@bnm.gov.my (T.T.); 2 Institute for Clinical Research, National Institutes of Health, Ministry of Health Malaysia, Setia Alam 40170, Malaysia; petertok.crc@gmail.com (P.S.K.T.); smongium1984@gmail.com (S.M.O.); masliyana@crc.gov.my (M.H.); sheamini@crc.gov.my (S.S.); drkalai@moh.gov.my (K.M.P.); 3 Disease Control Division, Ministry of Health Malaysia, Putrajaya 62590, Malaysia; mahesh@moh.gov.my (M.R.A.); dr_hazlina@moh.gov.my (H.Y.); dr_norhayati@moh.gov.my (N.R.); 4 Medical Development Division, Ministry of Health Malaysia, Putrajaya 62590, Malaysia; faizahmz@moh.gov.my (F.M.Z.); dr.shahanizan@moh.gov.my (S.M.Z.); drmdfikri@moh.gov.my (M.F.U.); 5 Office of Director-General, Ministry of Health Malaysia, Putrajaya 62590, Malaysia; tkpkpst@moh.gov.my (H.M.I.); anhisham@moh.gov.my (N.H.A.)

**Keywords:** COVID-19, SARS-CoV-2, COVID-19 vaccines, vaccine effectiveness, cohort study, Malaysia

## Abstract

Malaysia rolled out a diverse portfolio of predominantly three COVID-19 vaccines (AZD1222, BNT162b2, and CoronaVac) beginning 24 February 2021. We evaluated vaccine effectiveness with two methods, covering 1 April to 15 September 2021: (1) the screening method for COVID-19 (SARS-CoV-2) infection and symptomatic COVID-19; and (2) a retrospective cohort of confirmed COVID-19 cases for COVID-19 related ICU admission and death using logistic regression. The screening method estimated partial vaccination to be 48.8% effective (95% CI: 46.8, 50.7) against COVID-19 infection and 33.5% effective (95% CI: 31.6, 35.5) against symptomatic COVID-19. Full vaccination is estimated at 87.8% effective (95% CI: 85.8, 89.7) against COVID-19 infection and 85.4% effective (95% CI: 83.4, 87.3) against symptomatic COVID-19. Among the cohort of confirmed COVID-19 cases, partial vaccination with any of the three vaccines is estimated at 31.3% effective (95% CI: 28.5, 34.1) in preventing ICU admission, and 45.1% effective (95% CI: 42.6, 47.5) in preventing death. Full vaccination with any of the three vaccines is estimated at 79.1% effective (95% CI: 77.7, 80.4) in preventing ICU admission and 86.7% effective (95% CI: 85.7, 87.6) in preventing deaths. Our findings suggest that full vaccination with any of the three predominant vaccines (AZD1222, BNT162b2, and CoronaVac) in Malaysia has been highly effective in preventing COVID-19 infection, symptomatic COVID-19, COVID-19-related ICU admission, and death.

## 1. Introduction

Coronavirus disease 2019 (COVID-19) is a highly contagious disease caused by severe acute respiratory syndrome coronavirus 2 (SARS-CoV-2). Although COVID-19 principally targets the respiratory system, it can affect other major organ systems, potentially leading to death [1]. The main symptoms of COVID-19 include fever, cough, fatigue, and dyspnoea [2]. At present, the most used and validated diagnostic tests for COVID-19 include rapid antigen or antibody tests, immunoenzymatic serological tests, and molecular tests based on RT-PCR. Of these, RT-PCR-based molecular tests represent the gold standard in making a confirmatory diagnosis of COVID-19 infection [3]. Since being declared a pandemic on 11 March 2020, COVID-19 remains unresolved, with over 246 million confirmed cases and nearly five million deaths recorded by the end of October 2021 [4].

Given the immense public health cost from the ongoing coronavirus disease 2019 (COVID-19) pandemic, vaccines for severe acute respiratory syndrome coronavirus 2 (SARS-CoV-2) became crucial and were developed at an unprecedented pace [5]. Despite the short turnaround, clinical trials of now commonly administered SARS-CoV-2 vaccines registered efficacies against symptomatic COVID-19, hospitalisation, and COVID-19 related deaths above the WHO’s benchmark of 50% [6,7,8,9,10,11,12]. The AZD1222 (AstraZeneca) vaccine has a reported overall efficacy of 74.0% against symptomatic COVID-19 [8], while for the BNT162b2 (Pfizer-BioNTech) vaccine, efficacy measured 91.3% through six months of follow-up [10]. For the CoronaVac (Sinovac) vaccine, efficacy estimates are diverse. A phase 3 trial in Turkey reported an efficacy of 83.5% [9], while earlier estimates by national authorities were lower at 50.4% against symptomatic COVID-19 in Brazil and 65% in Indonesia [6]. AZD1222, BNT162b2, and CoronaVac are the three most administered vaccines in Malaysia.

With nationwide vaccination programmes being rolled out globally, evaluating vaccine efficacy becomes less feasible as trials do not reflect real-world conditions. Large observational studies, post licensure, to evaluate vaccine effectiveness in real-world settings are needed to complement findings from clinical trials [13,14]. Examples include large-scale studies to evaluate the effectiveness of BNT162b2 and AZD1222 in Israel and the United Kingdom [15,16,17,18] and of CoronaVac in Chile [13]. A broadly consistent finding is that vaccine efficacy is greater against severe disease than against infection and, at present, effective against severe disease from all main viral variants [19]. A notable gap is the lack of real-world evidence on vaccine effectiveness in low- and middle-income countries (LMICs) with logistic, demographic, and socio-economic conditions that differ from high-income countries (HICs), where most trials and effectiveness studies were conducted. Additionally, effectiveness estimates for CoronaVac remain limited, despite its dominance in many LMICs [20,21].

Malaysia has a population of 32.7 million in 2020, with 23.4 million aged 18 years and above [22,23]. As of 15 September 2021, Malaysia has reported over 2 million COVID-19 cases, becoming one of the most affected countries in the Western Pacific region [24]. Intensive public health measures and a nationwide lockdown were implemented in June 2021, while testing remained relatively extensive, considering increasingly limited hospital capacity and detection of new highly transmissible SARS-CoV-2 variants [25]. A critical step was, therefore, to implement effective vaccination strategies against COVID-19.

In Malaysia, the COVID-19 vaccines were administered through the National COVID-19 Immunisation Programme (*Programme Imunisasi COVID-19 Kebangsaan*; PICK), beginning 24 February 2021, over three phases [26]: phase 1 targeted ‘frontline’ workers; phase 2 prioritised ages 60 years and above, disabled, and high-risk individuals; phase 3 prioritised locations with high disease burden, and the remaining adult population. The phases in PICK considered epidemiological and clinical evidence, vaccine supply, and operational constraints. Nevertheless, demand constraints were not binding, as PICK’s registration rate consistently exceeded vaccine coverage and vaccination rates from 1 April 2021 to 15 September 2021 [22]. Appendix A and Appendix B detail Malaysia’s context within the COVID-19 pandemic, and PICK’s implementation, respectively.

As of 15 September 2021, 54.6% of Malaysia’s population is fully vaccinated. AZD1222 comprised 3.2 percentage points, BNT162b2 23.0 percentage points, and CoronaVac 26.5 percentage points. In PICK, homologous vaccines were administered for the first and second doses of the primary vaccination series for two-dose regimens. The diverse portfolio of vaccines used is an artefact of global vaccine inequity amid broadly lower supply in LMICs. Despite having advanced procurement agreements, most of Malaysia’s BNT162b2 supply was delivered in the late third quarter of 2021. This led to the wider use of CoronaVac, which comprised half of all completed vaccinations.

We evaluated vaccine effectiveness against COVID-19 (SARS-CoV-2) infections, symptomatic COVID-19, COVID-19 related intensive care unit (ICU) admissions, and COVID-19 related deaths for a diverse vaccine portfolio, which includes CoronaVac, in Malaysia. Over the course of PICK between 1 April 2021 and 15 September 2021, full vaccination with any of the three predominant vaccines (AZD1222, BNT162b2, and CoronaVac) has been highly effective in preventing COVID-19 infection, symptomatic COVID-19, COVID-19-related ICU admission, and death.

## 2. Materials and Methods

### 2.1. Data Environment

Absent of integrated electronic medical records, our analysis draws from four secondary data sets constructed from national COVID-19 surveillance: (1) the COVID-19 cases line listing, (2) the ICU admissions register, (3) the COVID-19 deaths line listing, and (4) the COVID-19 vaccine recipients line listing, linked deterministically with the case and personal identification numbers. At the time of writing, the COVID-19 cases and deaths line listings are published on the Ministry of Health Malaysia’s GitHub repository [27]. Reporting of COVID-19 cases and deaths is mandatory by law. Details on the definition of outcomes and data sources can be found in Appendix C and Appendix D, respectively.

Two methods are used: (1) the screening method to estimate vaccine effectiveness against COVID-19 (SARS-CoV-2) infections, symptomatic COVID-19, and (2) a retrospective cohort study for ICU admission and COVID-19 related deaths.

### 2.2. Screening Method Study Design, Population, and Methodology

The screening method was introduced by Orenstein et al. [28] to measure vaccine effectiveness, using aggregated data representative of the population. Recent studies that apply this method include the evaluation of vaccine effectiveness for COVID-19 infection and severe disease in Spain [29], for rotavirus in Japan [30], for the pneumococcal conjugate vaccine in the United States [31], and for influenza among the elderly in Germany [32]. We used national-level data spanning Malaysia’s population, comprising all individuals who have received at least one dose of the COVID-19 vaccines up to 15 September 2021, and confirmed COVID-19 cases between 1 April 2021 and 15 September 2021. Vaccine effectiveness and the confidence intervals are calculated as per Orenstein et al. [28]:

(1) $$VE_{Y}=100*\frac{\left(PPV_{d}-PCV_{Y,d}\right)}{PPV_{d}*\left(1-PCV_{Y,d}\right)}$$

The screening method estimates vaccine effectiveness (*VE*) against disease outcome *Y* (COVID-19 infection and symptomatic COVID-19) for the vaccination status *d* (partially or fully vaccinated, inclusive of all vaccine types) using the following variables, computed on a cumulative basis.

- Proportion of population that is vaccinated, *PPV*;
- Proportion of disease outcomes that are vaccinated, *PCV*.

### 2.3. Retrospective Cohort Study Design and Population

The retrospective cohort includes confirmed COVID-19 cases aged 18 years or older, assessed by reverse-transcriptase polymerase chain reaction assay (RT-PCR) or antigen testing. This reflects the initial age criteria to be vaccinated under PICK. Our cohort spans a six-month period from 1 April 2021 to 15 September 2021. Participants who received vaccines other than AZD1222, BNT162b2, and CoronaVac were excluded, as these vaccines were administered only towards the end of the study period and on a small number of people. Due to more complete information on the specific type of first dose received relative to that of the second dose, only information on the type of vaccine used for the first dose was used for analysis.

**Figure 1:**
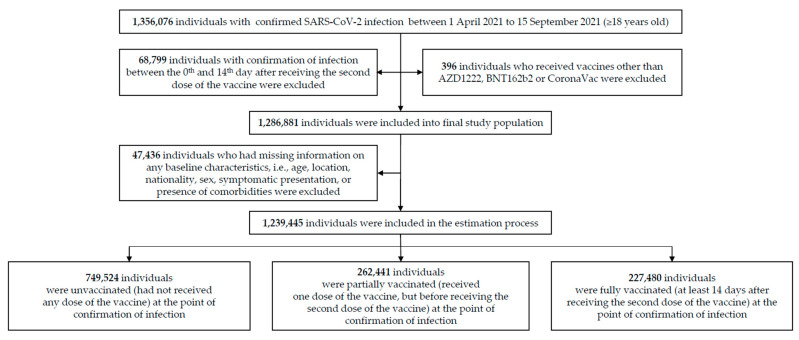
Study participants and cohort eligibility. Cohort participants were all confirmed SARS-CoV-2 cases in Malaysia from 1 April 2021 to 15 September 2021, aged 18 years or older. All participants either had not received any (unvaccinated) or at least one dose of the AZD1222, BNT162b2, or CoronaVac vaccines. Individuals who received any vaccines other than the ones specified above were excluded from the cohort.

Figure 1 depicts the study cohort. Of the 1,356,076 individuals aged 18 years or older with confirmed SARS-CoV-2 infections from 1 April 2021 to 15 September 2021, 1,286,881 were eligible for the study population. Study participants were classified into three groups: unvaccinated (did not receive any doses of AZD1222, BNT162b2, or CoronaVac), partially vaccinated (≥1 day after the receipt of the first dose of any of the three vaccines until before the receipt of the second dose), and fully vaccinated (≥14 days after the receipt of the second dose of any of the three vaccines).

We estimated vaccine effectiveness by vaccination status and type with logistic regression. This approach resembles the study by Hak et al. [33], who estimated adjusted odds ratios for severe symptoms or deaths among vaccinated and unvaccinated people from a cohort of influenza patients in the Netherlands.

Further description of the methodology is in Appendix E. All analyses were conducted with Python, version 3.9 [34].

## 3. Results

### 3.1. Screening Method: Vaccine Effectiveness

The screening method estimates partial vaccination to be 48.8% effective (95% CI: 46.8, 50.7) against COVID-19 infection and 33.5% effective (95% CI: 31.6, 35.5) against symptomatic COVID-19 (Table 1). Full vaccination is estimated to be 87.8% effective (95% CI: 85.8, 89.7) against COVID-19 infection and 85.4% effective (95% CI: 83.4, 87.3) against symptomatic COVID-19.

### 3.2. Cohort of Confirmed COVID-19 Cases: Vaccine Effectiveness

Table 2 presents descriptive statistics for the study cohort. A chi-squared test of independence suggests there are significant differences in the sex, age, nationality, symptomatic presentation, and presence of comorbidities according to vaccination status (unvaccinated, partially vaccinated, and fully vaccinated), as well as by vaccine type and vaccination status. A larger share of COVID-19 patients who were either partially or fully vaccinated was symptomatic at presentation. In contrast, most unvaccinated COVID-19 patients were asymptomatic at presentation. Further details are in Table A1.

**Figure 2:**
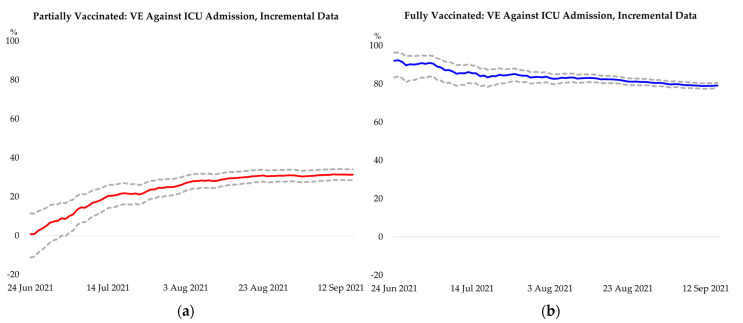

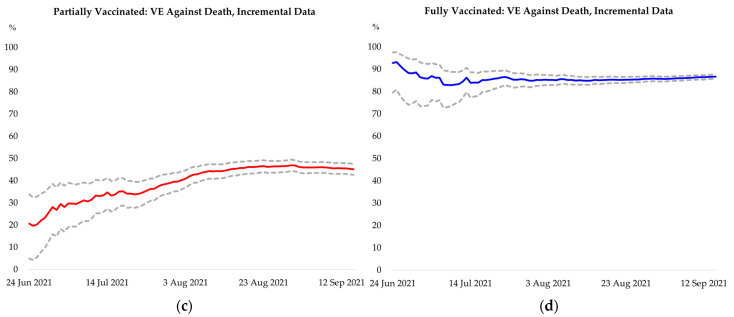
Robustness check for overall effectiveness of partial and full vaccination over the study period, with line plots representing the effectiveness for COVID-19-related ICU admission and death estimated with data from 1 April 2021 up to the corresponding day on the horizontal axis; dotted lines show the 95% confidence interval. (**a**) Effectiveness of partial vaccination against ICU admission; (**b**) Effectiveness of full vaccination against ICU admission; (**c**) Effectiveness of partial vaccination against death; (**d**) Effectiveness of full vaccination against death.

**Figure A2:**
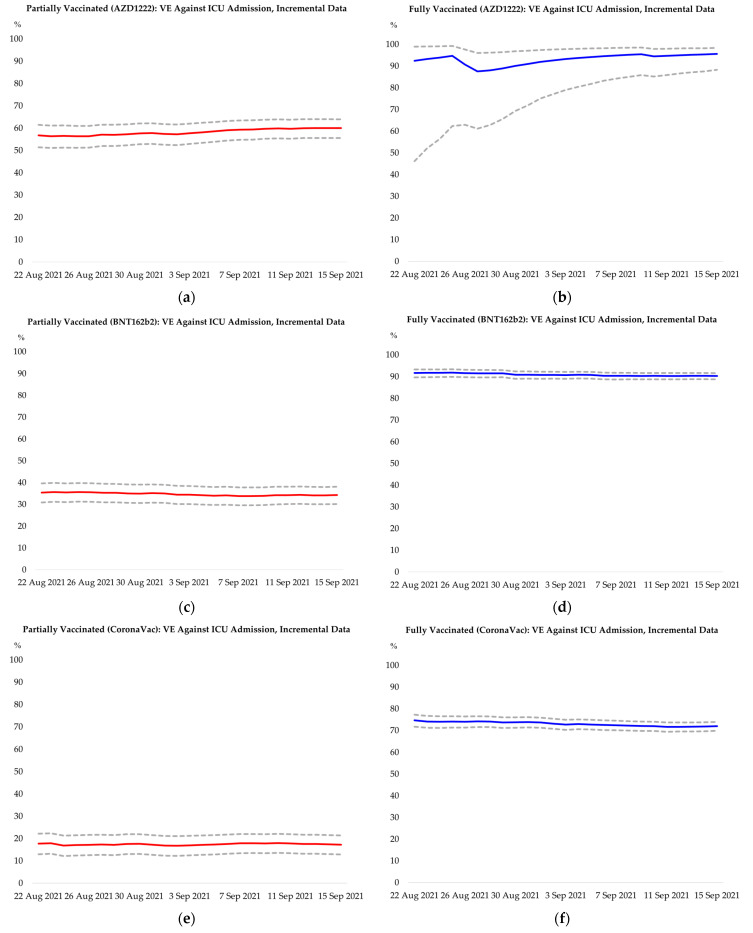
Robustness check for the overall effectiveness of partial and full vaccination by vaccine type over the study period, with line plots representing the effectiveness for COVID-19-related ICU admission estimated with data from 1 April 2021 up to the corresponding day on the horizontal axis; dotted lines show the 95% confidence interval. (**a**) Effectiveness of partial vaccination against ICU admission for AZD1222; (**b**) Effectiveness of full vaccination against ICU admission for AZD1222; (**c**) Effectiveness of partial vaccination against ICU admission for BNT162b2; (**d**) Effectiveness of full vaccination against ICU admission for BNT162b2; (**e**) Effectiveness of partial vaccination against ICU admission for CoronaVac; (**f**) Effectiveness of full vaccination against ICU admission for CoronaVac.

**Figure A3:**
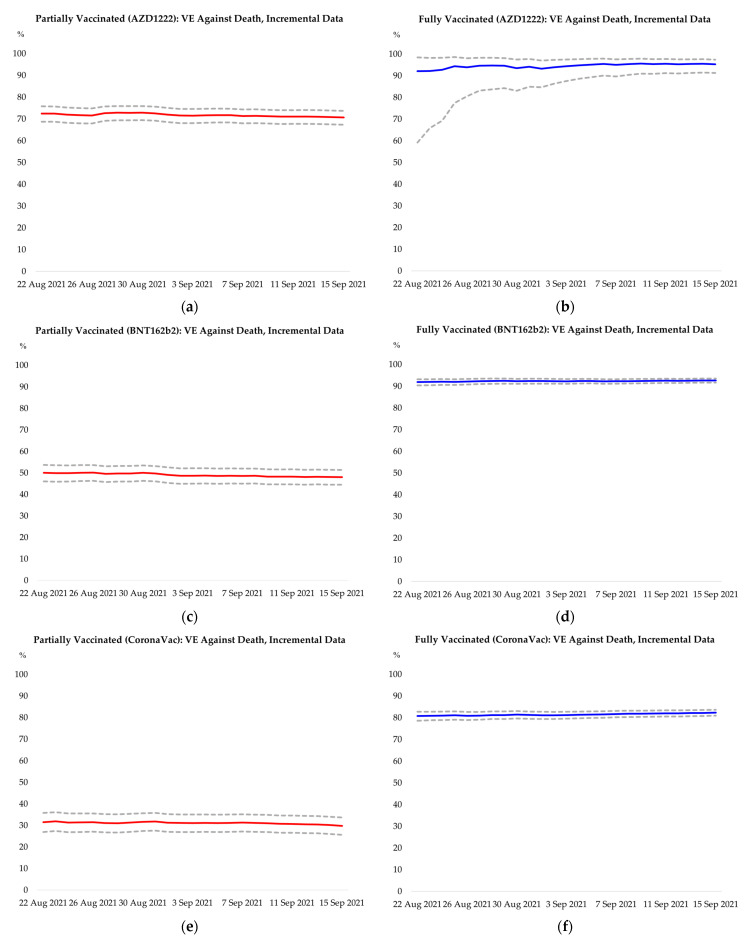
Robustness check for the overall effectiveness of partial and full vaccination by vaccine type over the study period, with line plots representing the effectiveness for COVID-19-related death estimated with data from 1 April 2021 up to the corresponding day on the horizontal axis; dotted lines show the 95% confidence interval. (**a**) Effectiveness of partial vaccination against death for AZD1222; (**b**) Effectiveness of full vaccination against death for AZD1222; (**c**) Effectiveness of partial vaccination against death for BNT162b2; (**d**) Effectiveness of full vaccination against death for BNT162b2; (**e**) Effectiveness of partial vaccination against death for CoronaVac; (**f**) Effectiveness of full vaccination against death for CoronaVac.

Table 3 presents vaccine effectiveness estimates in preventing admission to intensive care unit (ICU) and deaths among confirmed COVID-19 cases by vaccine type and vaccination status. Partial vaccination with any of the three vaccines is estimated to be 31.3% effective (95% CI: 28.5, 34.1) in preventing ICU admission and 45.1% effective (95% CI: 42.6, 47.5) in preventing deaths among confirmed COVID-19 cases. Full vaccination with any of the vaccines is estimated to be 79.1% effective (95% CI: 77.7, 80.4) in preventing ICU admission and 86.7% effective (95% CI: 85.7, 87.6) in preventing deaths among confirmed COVID-19 cases. Vaccine effectiveness estimates for partial and full vaccination, by the three vaccine types, are provided in Table 3. The unadjusted and partially adjusted estimates are in Table A2, Table A3, Table A4 and Table A5. In Table A6 and Table A7, we summarise the sensitivity of the fully adjusted, partially adjusted, and unadjusted effectiveness estimates by vaccine type to the definition of partial vaccination, considering both 1 day and 14 days after receiving the first dose of AZD1222, BNT162b2, or CoronaVac. In the alternative definition (≥14 days after the receipt of the first dose of any of the three vaccines), by excluding events occurring up to the 13th day after receiving the first dose, effectiveness for partial vaccination is higher than the main definition (≥1 day after the receipt of first dose), but the effectiveness estimates for full vaccination remain broadly unaffected. We further conduct a robustness check by estimating the effectiveness of partial and full vaccination with a daily-incremental sample and find the estimates to be stable over our study period (Figure 2, Figure A2, and Figure A3).

## 4. Discussion

This study presents findings on vaccine effectiveness in an LMIC with a uniquely diverse vaccine portfolio, primarily BNT162b2, AZD1222, and CoronaVac, which arose due to (1) setbacks in supply despite advance procurement agreements and broadly lower vaccine supply in LMICs, (2) maximisation of vaccine coverage under uncertainty in efficacy (limited evidence in early 2021), and (3) prioritisation of vulnerable groups amid the then-rising global prevalence of variants of concern, such as Delta (B.1.617.2). Despite these challenges in the nationwide rollout, as of 15 September 2021, more than half of Malaysia’s total population is fully vaccinated.

The findings show that being fully vaccinated reduced ICU admission with an effectiveness of 79.1% (95% CI: 77.7, 80.4) and death with an effectiveness of 86.7% (95% CI: 85.7, 87.6) among confirmed COVID-19 cases. Our estimates by vaccine types are comparable to interim estimates for Chile announced by the Government of Chile [35] in August 2021, which uses a similar portfolio of AZD1222, BNT162b2, and CoronaVac, albeit more reliant on CoronaVac. The complementary screening method estimated an effectiveness of 87.8% (95% CI: 85.8, 89.7) against COVID-19 infection and 85.4% (95% CI: 83.4, 87.3) against symptomatic COVID-19. This motivates that vaccines may reduce the risk of COVID-19 transmission and substantially for severe outcomes.

Using the screening method, our findings show that being fully vaccinated with any of the three vaccines studied reduces the risk of COVID-19 infections and symptomatic COVID-19 by at least 80%. These estimates are higher than those reported for preventing infections among elderly long-term care facility residents in Spain, where the screening method was used. This may be due to that long-term care facility residents having a higher exposure risk than the general population [29].

Real-world evidence on vaccine effectiveness for CoronaVac remains scarce, despite being approved by more than 30 countries and jurisdictions and administered in mass vaccination campaigns, particularly LMICs who experienced resurgences due to variants of concern [13,20,21,36]. Estimates of vaccine efficacy for CoronaVac against symptomatic COVID-19 in controlled trials varied from 51% to 84% [6,9]. A prospective national cohort study in Chile using the CoronaVac vaccine reported 87.5% effectiveness in preventing hospitalisation, 90.3% for preventing ICU admission, and 86.3% for preventing COVID-19-related death [13]. While the methodologies differ, our effectiveness estimates for CoronaVac in preventing ICU admission (72.0%) and deaths (82.4%) add to existing evidence that full vaccination with CoronaVac is highly effective in preventing severe outcomes due to COVID-19.

Our study notes that the effectiveness estimates for the AZD1222 and BNT162b2 vaccines are higher than that of the CoronaVac vaccine. The effectiveness estimates of both vaccines for preventing ICU admission and death are comparable to earlier studies, although methodological differences exist [19]. The estimates for AZD1222 may be subject to selection bias due to its opt-in nature, while its later rollout resulted in a shorter follow-up period than the other two vaccines. Nonetheless, the estimates are robust to varying the time coverage of the study cohort (Figure 2), which spanned a period coinciding with the prevalence of new variants of concern [37,38,39]. The estimates do not uncover vaccine effectiveness to specific variants, given insufficient granular data on the breakdown of the retrospective cohort participants by specific variants. To robustly assess this aspect, more comprehensive genomic surveillance needs to be conducted.

Our findings should be interpreted with two caveats. First, effectiveness against COVID-19 infections and symptomatic COVID-19 are estimated with aggregate data and the screening method. This trades away adjustments for confounders and is subject to an upward bias under non-comprehensive testing [28]. While computationally simple and feasible with aggregate data, the methodology could not explicitly account for stratification by specific vaccine types. In contrast, the logistic regression in the retrospective cohort approach allows this flexibility. Given this and the differently timed rollouts of the three vaccines, it may be unfeasible to estimate effectiveness by vaccine types. Hence, we caution on generalising the estimates, despite being comparable to previous studies, which investigated the effectiveness of the three dominant vaccines in Malaysia, albeit via different methods [9,13,15,19].

Second, adequate visibility at the individual level was limited to confirmed COVID-19 cases. Hence, effectiveness against ICU admission and death are conditional on infection. As we analysed available secondary data sets in the retrospective cohort approach, we were limited by the availability of variables and were unable to investigate socio-economic factors or related risk behaviours. Moreover, only the presence of symptoms and comorbidities could be ascertained, but estimates do not change materially based on sensitivity analyses. Finally, our study period of over five months may be inadequate to observe all outcomes of interest for cases near the end of the study period.

Our study has three strengths. First, we used a rich data set consolidated from multiple official and granular data sources with nationally representative coverage. This enabled controlling for confounders at the individual level within our cohort of confirmed COVID-19 cases. Second, the study was conducted when COVID-19 incidence rates in Malaysia peaked and spanned the emergence of many SARS-CoV-2 variants. This enhances the reliability of our effectiveness estimates amid uncertainty over the impact of these variants on the effectiveness of widely administered vaccines [15,37,38]. While we cannot ascertain the impact of specific variants of concern due to the lack of genomic data at the individual level, we demonstrated that effectiveness estimates are stable over the latter part of our study period. Third, our study addresses the gap in evidence on vaccine effectiveness in LMICs and contributes insights on the effectiveness of a diverse vaccine portfolio within a nationwide mass vaccination programme.

## 5. Conclusions

Our findings show that the COVID-19 vaccines used in Malaysia are effective, particularly in preventing ICU admission and death among COVID-19 cases, consistent with other studies. At present, we are unable to report detailed results on the safety profiles and adverse events of vaccines under PICK, for which monitoring and evaluation are ongoing. As current cross-country evidence [19], including our findings, show that vaccine effectiveness against severe outcomes remains high, further research is needed to ascertain the optimal timing for boosting in the general population.

## Figures and Tables

**Table 1:** Proportion of COVID-19 cases vaccinated and estimates of vaccine effectiveness using the screening method.

	PCV (%)	PPV (%)	VE (%)	95% CI
**COVID-19 (SARS-CoV-2) Infection**				
Partial Vaccination	16.7	28.2	48.8	46.8, 50.7
Full Vaccination	16.6	62.0	87.8	85.8, 89.7
**Symptomatic COVID-19**				
Partial Vaccination	20.7	28.2	33.5	31.6, 35.5
Full Vaccination	19.2	62.0	85.4	83.4, 87.3

Abbreviations: CI, confidence intervals; COVID-19, coronavirus disease; PCV, proportion cases vaccinated; PPV, proportion population vaccinated.

**Table 2:** Characteristics of the study cohort of confirmed COVID-19 cases, according to vaccination status.

Characteristic	Cohort		Unvaccinated		Partially Vaccinated		Fully Vaccinated		*p*-Value
	*n*	%	*n*	%	*n*	%	*n*	%	
Participants no.	1,286,881	100.0	788,464	61.3	269,528	20.9	228,889	17.8	
AZD1222	50,423	3.9			45,736	3.6	4687	0.4	
BNT162b2	176,600	13.7			92,677	7.2	83,923	6.5	
CoronaVac	271,394	21.1			131,115	10.2	140,279	10.9	
**Sex**									<0.001
Female	551,330	42.8	317,937	40.3	121,854	45.2	111,539	48.7	
Male	735,551	57.2	470,527	59.7	147,674	54.8	117,350	51.3	
**Age group**									<0.001
Below 20	49,217	3.8	35,447	4.5	9524	3.5	4246	1.9	
20 to 29	381,826	29.7	248,752	31.5	83,091	30.8	49,983	21.8	
30 to 39	332,779	25.9	207,440	26.3	69,616	25.8	55,723	24.3	
40 to 49	205,691	16.0	123,544	15.7	45,413	16.8	36,734	16.0	
50 to 59	139,563	10.8	71,931	9.1	30,527	11.3	37,105	16.2	
60 to 69	84,915	6.6	40,096	5.1	16,712	6.2	28,107	12.3	
70 to 79	33,563	2.6	15,687	2.0	5661	2.1	12,215	5.3	
80 and above	11,891	0.9	6627	0.8	1897	0.7	3367	1.5	
*Missing*	47,436	3.7	38,940	4.9	7087	2.6	1409	0.6	
**Nationality**									<0.001
Malaysian	1,038,080	80.7	587,240	74.5	229,917	85.3	220,923	96.5	
Non-Malaysian	248,801	19.3	201,224	25.5	39,611	14.7	7966	3.5	
**Presentation of Symptoms**									<0.001
Asymptomatic	647,740	50.3	426,377	54.1	115,122	42.7	106,241	46.4	
Symptomatic	639,141	49.7	362,087	45.9	154,406	57.3	122,648	53.6	
**Presence of Comorbidities**									<0.001
No comorbidities	1032,442	80.2	622,953	79.0	216,205	80.2	193,284	84.4	
Comorbid	254,439	19.8	165,511	21.0	53,323	19.8	35,605	15.6	

Abbreviations: AZD1222, Oxford-AstraZeneca; BNT162b2, Pfizer-BioNTech; CoronaVac, Sinovac.

**Table 3:** Vaccine effectiveness in preventing admission to ICU and deaths among COVID-19 cases, according to types of vaccines and vaccination status.

Outcomes and Vaccine Effectiveness	Cohort of Confirmed COVID-19 Cases			Vaccine Effectiveness (95% CI)							
	Total No.	Event No.	Rate of Event	All Types		AZD1222		BNT162b2		CoronaVac	
			(per 1000 Persons)	VE	95% CI	VE	95% CI	VE	95% CI	VE	95% CI
**Admission to ICU**											
Unvaccinated	749,524	14,497	19.3	ref	ref	ref	ref	ref	ref	ref	ref
Partially vaccinated	262,441	3715	14.2	31.3	28.5, 34.1	60.0	55.6, 64	34.3	30.2, 38.1	17.3	12.9, 21.4
Fully vaccinated	227,480	1156	5.1	79.1	77.7, 80.4	95.6	88.3, 98.4	90.3	88.8, 91.6	72.0	69.9, 73.9
**Confirmed death**											
Unvaccinated	749,524	15,976	21.3	ref	ref	ref	ref	ref	ref	ref	ref
Partially vaccinated	262,441	4766	18.2	45.1	42.6, 47.5	70.7	67.3, 73.7	48.1	44.5, 51.4	29.8	25.7, 33.7
Fully vaccinated	227,480	1601	7.0	86.7	85.7, 87.6	95.3	91.3, 97.4	92.7	91.7, 93.6	82.4	81.0, 83.7

Adjusted for age, presence of comorbidities, presentation of symptoms, state dummies, and a linear trend term (epidemic day since the start of the cohort). Abbreviations: CI, confidence intervals; COVID-19, coronavirus disease; ICU, intensive care unit; VE, vaccine effectiveness; AZD1222, Oxford-AstraZeneca; BNT162b2, Pfizer-BioNTech; CoronaVac, Sinovac; ref, reference.

**Figure A1:**
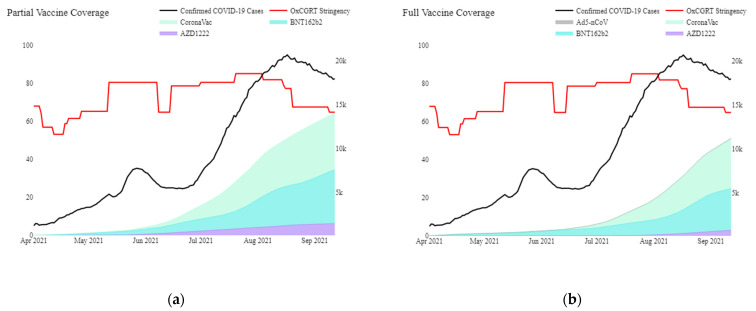
COVID-19 vaccine coverage, daily new confirmed COVID-19 cases, and the OxCGRT Stringency Index in Malaysia from 1 April 2021 to 15 September 2021. (**a**) Partially vaccinated; (**b**) fully vaccinated.

## Data Availability

The anonymised data presented in this study are available on reasonable request.

## Appendix A. Additional Context on Malaysia (Setting) and COVID-19 Pandemic

Malaysia has a reported population of 32.7 million in 2020, of whom approximately 29.7 million are citizens and 3.0 million are non-citizens. Malaysia has a multi-ethnic population. The three largest ethnicities are the Malays (69.6%), Chinese (22.6%), and Indians (6.8%) [23]. About 23.4 million of Malaysia’s population are aged 18 years and above, while 3.5 million are aged 60 years and older [22,23].

Malaysia has seen three waves of COVID-19 since reporting its first case on 4 February 2020, followed by a resurgence driven by local transmission from a large-scale social gathering, culminating in a nationwide lockdown on 18 March 2020. After daily cases remained low at single and double digits between July to September 2020, Malaysia experienced its third wave in October 2020. Both cases and deaths remained elevated [40,41]. In May 2021, reported COVID-19 cases rose peaked at 8,000 daily, with more than 70,000 active cases at one time. Taking into consideration the rise in cases, healthcare response burden (increasingly limited hospital capacity, quarantine units, and test capacity to treat COVID-19 patients), as well as an increase in the prevalence of variants of concern, especially Beta (B.1.351) and Delta (B.1.617.2), a nationwide lockdown was implemented from 1 June 2021 onwards, restricting economic activities to essential and export-oriented sectors, while all social activities and inter-district movement were prohibited [25,42].

Malaysia has a high burden of non-communicable diseases. In the recent national health and morbidity survey (NHMS) conducted in 2019, an overall prevalence of 18.3% of diabetes and 30.0% of hypertension among adults was reported [43]. About one in every two adults in Malaysia was found to be overweight or obese (30.4% overweight and 19.7% obese), and 21.3% of adults were current smokers [43]. These risk factors have been associated with a higher risk of severe illness from COVID-19 [44], thus warranting further attention amid the rise in COVID-19 infections.

Along with movement, and economic restrictions implemented during the lockdown, intensive public health measures were also executed to bring the surge of cases under control. These included increased genomic surveillance, expanded capacities at quarantine centres, hospitals, and intensive care units (ICUs), and reprioritised healthcare resources to outbreak areas facing surges in COVID-19 cases. Even as vaccines were rolled out, throughout PICK, COVID-19 testing remained extensive in Malaysia, and was among the highest globally. Between 1 April 2021 and 15 September, Malaysia conducted a total of 18,227,550 COVID-19 diagnostic tests, or 55.8 tests per 100 population (RTK-Ag: 8,526,783 or 26.1 per 100 population; RT-PCR: 9,700,767 or 29.7 per 100 population), one of the highest globally, including high-income countries (HICs). The average positivity rate was 7.97% [23,42]. Finally, data analytics were also intensified to assist the epidemic management effort, leading to the creation of automated contact tracing and hotspot prediction systems using granular movement and COVID-19 diagnostic test data.

## Appendix B. Programme Imunisasi COVID-19 Kebangsaan (PICK)

The Government of Malaysia’s overarching vaccination strategy is to maximise vaccine coverage in the shortest timespan feasible to minimise deaths and severe COVID-19 outcomes. COVID-19 vaccines are provided for free to all residents, including non-citizens, of Malaysia. To ensure timely, safe, and effective access to COVID-19 vaccines, the Special Committee for Ensuring Access to COVID-19 Vaccine Supply (*Jawatankuasa Khas Jaminan Akses Bekalan Vaksin COVID-19*, JKJAV) was established [26]. JKJAV, co-chaired by the Ministry of Health and the Ministry of Science, Technology and Innovation, is responsible for the planning, implementation, and monitoring of the National COVID-19 Immunisation Programme (*Programme Imunisasi COVID-19 Kebangsaan*, PICK). JKJAV decides medical-related policies and issues, such as the choice of vaccine and vaccination for adolescents. The coordination, and execution of PICK, rests on the COVID-19 Immunisation Task Force (CITF), set up by the executive arm of the Government of Malaysia. Focus areas often involve other ministries, such as the Ministry of International Trade and Industry (MITI), to coordinate demand from manufacturers and the Ministry of Youth and Sports to secure volunteers.

COVID-19 vaccines in Malaysia were administered through PICK, beginning 24 February 2021, over three major phases [26]:

- Phase 1 targeted ‘frontline’ workers, mostly in the healthcare, defence, and security sectors, involving approximately 500,000 individuals (1.5% of the population);
- Phase 2, which began in April 2021, prioritised senior (aged 60 years and above), disabled and high-risk individuals for severe diseases. Phase 2 involved approximately 9.4 million individuals (28.8% of the population);
- Phase 3 involved the remaining population aged 18 years and above and prioritises locations with high disease burdens to minimise the adverse effects of COVID-19.

Overall, PICK’s prioritisation strategy carefully considered epidemiological and clinical evidence, the availability of vaccines, and operational constraints.

As of February 2021, up to 66.7 million doses of COVID-19 vaccines were secured through the COVID-19 Vaccines Global Access (COVAX) Facility and advanced purchases from five manufacturers9. The supply and delivery of vaccines were phased and approved by the National Pharmaceutical Regulatory Agency (NPRA) before being rolled out. BNT162b2 was the first vaccine to be conditionally approved on 8 January 2021, followed by CoronaVac and AZD1222 on 2 March 2021 [45]. Other vaccines have also been conditionally approved by the NPRA, although BNT162b2, AZD1222, and CoronaVac constituted the majority of vaccines administered. Since the beginning of PICK up until 15 September 2021, all three vaccines were two-dose regimes, with doses administered 21 days apart for BNT162b2 and CoronaVac and 63 days apart for AZD1222. An individual is deemed fully vaccinated 14 days after receiving the second dose of any of the three vaccines (BNT162b2, CoronaVac, and AZD1222).

Mass vaccination drives globally are often met with logistical challenges, including delivery uncertainty, storage and administration of vaccines, the quality and capacity of healthcare infrastructure, as well as demographics. Malaysia faced similar challenges, and selective rollout of vaccines was often necessary dependent on the factors mentioned above. BNT162b2 was primarily administered in Phases 1 and 3 of PICK. CoronaVac was administered primarily in phase 2 and the early part of phase 3. AZD1222 was introduced in May 2021 through two opt-in registrations, focused on areas with high disease burdens, such as Klang Valley (Selangor, Kuala Lumpur, and Putrajaya), Kuching District of Sarawak, Penang, and Johor, before being fully integrated into PICK by July 2021.

There were certain unique circumstances surrounding the rollout of the respective vaccines under PICK. CoronaVac was conditionally approved for the general population on 2 March 2021, prior to the WHO’s authorisation for emergency use on 1 June 2021, amid early evidence of adequate efficacy [6,9]. The initial plan to roll out AZD1222 in May 2021 was met with hesitancy due to international reports linking the vaccine to thrombosis with thrombocytopenia syndrome (TTS). CITF introduced a separate opt-in stream for the AZD1222 vaccine after being approved by JKJAV for use. Following two opt-in launches in May 2021, which recorded overwhelming demand, AZD1222 was subsequently reintegrated into the mainstream rollout under PICK. This deviation from the initially planned phases of PICK is reflected in generally younger recipients for AZD1222, who had voluntarily signed up to receive vaccinations earlier in lieu of waiting for phase 3.

Given that epidemic control was an objective of PICK, the timing and resources dedicated to vaccination were reprioritised and differed across states during Phases 2 and 3. Vaccination rates in states with worse COVID-19 disease burden would be higher, exerting biases on the vaccine effectiveness estimates if left unadjusted. Hence, our analysis adjusted for state-specific factors with state dummies.

PICK was ramped up in July 2021 when supply constraints were resolved, which coincided with the peak of the national infection incidence rate and increasing prevalence of variants of concern. Cumulative confirmed COVID-19 cases exceeded one million on 26 July 2021 and eclipsed two million by 27 September 2021. To facilitate a rapid rollout, PICK leveraged on private healthcare providers to supplement mega vaccination centres, as well as outreach programmes to vaccinate individuals living in remote areas, especially in the states of Sabah and Sarawak. In essence, PICK remobilised national resources to maximise vaccine coverage over the shortest possible duration to mitigate COVID-19 amid early supply constraints, as well as uncertainty over the then-available vaccines.

The public healthcare system and infrastructure in Malaysia were pivotal in facilitating the rapid rollout of PICK. Immunisation programmes in Malaysia could be traced to their integration within the Maternal and Child Health (MCH) programmes since the 1950s. This network of healthcare resources, remobilised into a dense network of vaccination centres and outreach programmes, facilitated a rapid rollout of vaccines nationwide, including rural areas.

Underpinning PICK’s demand and rapid rollout is a digitalised, user-friendly process that is integrated into the Malaysia Vaccine Administration System (MyVAS). The entire flow from registration, scheduling of appointments, validation of vaccination, monitoring of adverse events following immunisation (AEFIs) could be conducted through the MySejahtera application. Individuals without the MySejahtera application could register as dependents of pre-existing MySejahtera users at designated public and private healthcare facilities, a dedicated telephone hotline, the PICK website. Importantly, communities in rural, interior, or hard-to-reach areas were vaccinated via outreach programmes upon consent, even without prior registration. Appointments were optimised for accessibility by vaccine registrants and reflective of the prioritisation of the various phases of PICK.

High vaccine effectiveness is reflected in Malaysia’s improved state of the epidemic. COVID-19 deaths and ICU admissions trended down after peaking in July by September, in line with observations in HICs with high vaccine coverage but less diverse portfolios, e.g., Singapore and the United Kingdom. This reduction in healthcare burden facilitated a gradual lifting of restrictions on economic sectors. Malaysia’s vaccination strategy, which prioritised maximum and rapid vaccine coverage amid supply constraints, uncertainty in the effectiveness of then-available vaccines, and rising prevalence of new variants, can be a reference, especially supply-constrained LMICs with low vaccine coverage. Importantly, PICK’s logistic success, reflected in vaccine coverage exceeding most HICs within a short duration [46], rests on robust computerised, centralised, and easily analysable COVID-19 databases.

As of 15 September 2021, through PICK, the proportion of the population fully vaccinated in Malaysia is at 54.6%, of which AZD1222 comprised 3.2 percentage points, BNT162b2 23.0 percentage points, and CoronaVac 26.5 percentage points. Additionally, beginning September 2021, the vaccination of adolescents (aged 12 to 17 years) in Malaysia commenced with the BNT162b2 vaccine. Figure A1 shows Malaysia’s vaccine coverage by type, confirmed cases, and the Oxford COVID-19 Government Response Tracker (OxCGRT) Stringency Index [47], which indicates containment restrictiveness from 1 April 2021 to 15 September.

## Appendix C. Definition of Outcome Measures

Definition of outcome measures is based on the COVID-19 management guidelines by the Ministry of Health Malaysia.

- COVID-19 confirmed cases
  A COVID-19 confirmed case was defined as a person with laboratory confirmation* of infection with the COVID-19 irrespective of clinical signs or symptoms.
  *A person with a positive molecular test (RT-PCR or rapid molecular) or a person with positive RTK-Ag in pre-determined areas/locality with the prevalence of COVID-19 ≥ 10%
- Symptomatic COVID-19 confirmed cases
  A COVID-19 confirmed case with reported signs or symptoms such as fever, chill, rigours, myalgia, headache, sore throat, nausea or vomiting, diarrhoea, fatigue, nasal congestion, cough, shortness of breath, difficulty in breathing, sudden onset of anosmia, and/or ageusia.
- COVID-19 ICU admissions
  A confirmed COVID-19 case that required intensive care and was admitted to ICU based on clinical judgement due to disease progression to severe diseases, such as category 4 (requiring oxygen therapy) or category 5 (requiring mechanical ventilation).
- COVID-19 deaths
  A COVID-19 death refers to any “death attributable to COVID-19” and took two settings as follows:
  - Criteria for death during hospitalisation:
    - COVID-19-positive case that has been confirmed through laboratory tests (RT-PCR)
    - Death resulting from clinically compatible disease
    - No clear alternative cause of death that is not related to COVID-19 infection (examples: death due to trauma, road traffic accident, suicide, etc.)
    - No period of complete recovery from COVID-19 between illness and death
    - Even with pre-existing disease (e.g., cancer), it is considered death due to COVID-19 if the reason for the severe course is due to COVID-19 infection
  - Criteria for cases that were brought in dead (BID):
    - If a postmortem is conducted, the classification of COVID-19 death shall be in accordance with the autopsy death report;
    - If the autopsy is not performed, the death of COVID-19 must meet the following criteria:
      - COVID-19-positive case that has been confirmed through laboratory test (RT-PCR) with CT value; and
      - Has an epidemiological link with another COVID-19 case.
    - Supportive radiological investigation, if any (examples: chest x-ray, postmortem computed tomography scan, etc.), which shows signs of COVID-19 infection, can also be used as a supportive modality for the attending specialist in charge. However, it is not a criterion that must be met for the confirmation of “Death Due to COVID-19”.

## Appendix D. Data Sources

National-level data were extracted from secondary data sources from the surveillance system at the National Crisis Preparedness and Response Centre (CPRC) under the Ministry of Health (MOH) Malaysia. COVID-19 surveillance data are captured from both public and private healthcare facilities based on the provision of the Prevention and Control of Infectious Diseases Act 1988 (Act 342).

- The COVID-19 cases line listing
  Data source: Malaysia national electronic COVID-19 cases register (e-COVID/COVID-19 line listing).
  Data on the COVID-19 confirmed cases were obtained from the national electronic COVID-19 cases register, which is the national COVID-19 surveillance system. This surveillance system detects cases from multiple sources such as the surveillance system, point of entry, targeted screening, international health regulation focal points, pre-admission of COVID-19 screening, passive case detection from healthcare facilities, and any screening for brought-in dead. Each patient diagnosed as a confirmed case will be assigned a unique case number. Data elements include patient demographics, location, date of positive test, presence of comorbidities, and symptoms at diagnosis.
- The intensive care unit (ICU) admissions register
  Data source: Malaysia national COVID-19 clinical admissions register
  Data on ICU admissions for COVID-19 patients were obtained from the National COVID-19 clinical admission register. This is an administrative register collecting information on allCOVID-19 ICU admissions containing elements such as patient demographic, location, symptoms, disease category at admission, and comorbidities.
- The COVID-19 deaths line listing
  Data source: Malaysia national COVID-19 deaths line listing
  The criteria for the classification of COVID-19 death have been developed and established by a consensus from Infectious Diseases Physicians, Forensic Pathologists, and the National Committee of COVID-19 Mortality Review. Every death underwent an audit process based on the WHO guidelines before it was classified as COVID-19 death. Data elements included patient demographics, location, date of death, and comorbidities.
- The COVID-19 cases line listing
  Data source: Malaysia national COVID-19 vaccinations register
  Vaccination records for the Malaysian population were obtained from the National COVID-19 vaccination register, available via the Vaccine Management System of the Ministry of Health Malaysia (Malaysia Vaccine Administration System, MyVAS).
  Data elements included patient-level demographics and comorbidities, date of vaccination, site of vaccination, vaccine batch numbers, and vaccine type. Data fields pertaining to background characteristics were self-reported when patients registered for the COVID-19 vaccine via PICK, while data fields on vaccination were reported by healthcare providers at vaccination centres via MyVAS. Verification steps were built in. Firstly, selected workers at the vaccination centres with administrator rights verified the validity of data fields for respective patients via MyVAS, including the actual administration of vaccines and demographics. Secondly, patients were required to scan QR codes at various stations to validate the vaccine administration through Malaysia’s contact tracing application, MySejahtera. For non-MySejahtera users, workers at the vaccination centres perform the validation via MyVAS.

The registry encompassed all individuals who have received a COVID-19 vaccine via PICK and is the ‘single source of truth’ of COVID-19 vaccine recipients in Malaysia. The data were constructed for the planning and operationalisation of PICK and were directly used in issuing appointments, tracking vaccine coverage by demographics, risk groups, and location, as well as in planning the allocation of resources.

## Appendix E. Methodology

### Appendix E.1. Screening Method

Vaccine effectiveness is calculated as follows, with confidence intervals as per Orenstein et al. [28]:

(A1) $$VE_{Y,i}=100*\frac{\left(PPV_{d}-PCV_{Y,d}\right)}{PPV_{d}*\left(1-PCV_{Y,d}\right)}$$

(A2) $$CI_{95\%}=1-\left(\left(1-VE_{Y,i}\right)\;\pm 1.96*\sqrt{\frac{1-\frac{CV_{Y,d}}{V_{d}}}{CV_{Y,d}}+\frac{1-\frac{CnV_{Y}}{nV}}{CnV_{Y}}}\right)$$

The screening method estimates vaccine effectiveness (*VE*) against disease outcome *Y* (infection and symptomatic infection) for the vaccination status *d* (partially or fully vaccinated, inclusive of all vaccine types) using the variables as follows, computed on a cumulative basis.

- The proportion of the population that is vaccinated, or vaccination coverage in population, *PPV*.
- The proportion of outcomes (infections, or symptomatic infections) that are vaccinated, *PCV*, since 1 April 2021.
- The number of outcomes that are vaccinated, *CV*, since 1 April 2021.
- The number of outcomes that are unvaccinated, *CnV*, since 1 April 2021.
- The number of vaccinated individuals, *V*.
- The number of unvaccinated individuals, *nV*.

This approach compares the observed gap between vaccine coverage (*PPV*) and the proportion of outcomes (*PCV*) that are vaccinated against an unobserved counterfactual of 100% vaccine effectiveness. While computationally simple, the screening method does not permit stratification by vaccine types, as the *PPV* term will be constrained to values less than 1, as opposed to freely between 0 (zero vaccine coverage) and 1 (full vaccine coverage), due to the rollout of multiple vaccine types, otherwise resulting in potentially incorrect effectiveness estimates.

### Appendix E.2. Retrospective Cohort: Logistic Regression

Drawing from the individual-level data sets, this empirical strategy estimates the adjusted odds ratio against ICU admission and death, conditional on age, presence of comorbidities, presentation of symptoms, state- and time-specific unobservables, for the retrospective cohort of COVID-19 cases. The vaccine effectiveness is implied directly by the estimated adjusted odds ratios.

Absent of individual-level data on demographic factors, location, comorbidities on individuals who test negative, i.e., not infected by COVID-19, we cannot infer effectiveness as in a traditional cohort or case-control study, which includes uninfected individuals. However, this provides a clean interpretation on risk reduction in severe outcomes, and deaths, assuming infected.

(A3) $$\mathrm{Pr}{\left(Y\right)}_{i}=\Lambda \left(\alpha +{\left\{Vax\right\}}_{Dose,i}^{\left(Type\right)}\cdot \beta_{Dose,i}^{\left(Type\right)}+X_{i}\cdot \lambda +\epsilon_{i}\right)$$

(A4) $${\left\{VE_{Y}\right\}}_{Dose}^{Type}=100*\left(1-\exp\left(\beta_{Y}{}_{Dose}^{\left(Type\right)}\right)\right)$$

The model is linearly parameterised. We regress the disease outcome of interest *Y* (ICU admission and death) on a constant, a dummy indicating the individual’s vaccination status (partially, and fully, with the unvaccinated group being the omitted term), {*Vax*}, which is disaggregated further into AZD1222, BNT162b2, and CoronaVac. The vaccine effectiveness is then calculated from the adjusted odds ratio on the ‘treatment’ dummy via 100 × (1 − exp(***β***)). We included a vector of controls in ***X***, consisting of age, age-squared, a dummy indicating the presence of comorbidities, symptomatic presentation, nationality dummy, gender dummy, state dummies, and a linear trend term (epidemic day since the start of the cohort). Age enters the model as a continuous term, as opposed to the more commonly used age group dummies, and the squared term allows for nonlinearity in the effect of age on the odds of ICU admission or death.

## Appendix F. Additional Results

**Table A1:** Characteristics of the study cohort of confirmed COVID-19 cases, according to the type of vaccines and vaccination status.

Characteristic	Cohort		Unvaccinated		Partially Vaccinated: AZD1222		Fully Vaccinated: AZD1222		Partially Vaccinated: BNT162b2		Fully Vaccinated: BNT162b2		Partially Vaccinated: CoronaVac		Fully Vaccinated: CoronaVac		*p*-Value
	*n*	%	*n*	%	*n*	%	n	%	n	%	*n*	%	*n*	%	*n*	%	
Participants no.	1,286,881	100.0	788,464	61.3	45,736	3.6	4687	0.4	92,677	7.2	83,923	6.5	131,115	10.2	140,279	10.9	
AZD1222	50,423	3.9			45,736	3.6	4687	0.4									
BNT162b2	176,600	13.7							92,677	7.2	83,923	6.5					
CoronaVac	271,394	21.1											131,115	10.2	140,279	10.9	
**Sex**																	<0.001
Female	551,330	42.8	317,937	40.3	22,819	49.9	2218	47.3	45,891	49.5	43,378	51.7	53,144	40.5	65,943	47.0	
Male	735,551	57.2	470,527	59.7	22,917	50.1	2469	52.7	46,786	50.5	40,545	48.3	77,971	59.5	74,336	53.0	
**Age group**																	<0.001
Below 20	49,217	3.8	35,447	4.5	1440	3.1	77	1.6	3807	4.1	793	0.9	4277	3.3	3376	2.4	
20 to 29	381,826	29.7	248,752	31.5	14,854	32.5	1474	31.4	28,048	30.3	17,449	20.8	40,189	30.7	31,060	22.1	
30 to 39	332,779	25.9	207,440	26.3	11,630	25.4	1038	22.1	23,331	25.2	24,790	29.5	34,655	26.4	29,895	21.3	
40 to 49	205,691	16.0	123,544	15.7	6903	15.1	537	11.5	15,291	16.5	12,933	15.4	23,219	17.7	23,264	16.6	
50 to 59	139,563	10.8	71,931	9.1	4324	9.5	385	8.2	12,544	13.5	11,843	14.1	13,659	10.4	24,877	17.7	
60 to 69	84,915	6.6	40,096	5.1	4662	10.2	887	18.9	5617	6.1	9178	10.9	6433	4.9	18,042	12.9	
70 to 79	33,563	2.6	15,687	2.0	1295	2.8	207	4.4	2333	2.5	5312	6.3	2033	1.6	6696	4.8	
80 and above	11,891	0.9	6627	0.8	441	1.0	72	1.5	822	0.9	1482	1.8	634	0.5	1813	1.3	
*Missing*	47,436	3.7	38,940	4.9	187	0.4	10	0.2	884	1.0	143	0.2	6016	4.6	1256	0.9	
**Nationality**																	<0.001
Malaysian	1,038,080	80.7	587,240	74.5	43,704	95.6	4571	97.5	85,103	91.8	82,861	98.7	101,110	77.1	133,491	95.2	
Non-Malaysian	248,801	19.3	201,224	25.5	2032	4.4	116	2.5	7574	8.2	1062	1.3	30,005	22.9	6788	4.8	
**Presentation of Symptoms**																	<0.001
Asymptomatic	647,740	50.3	426,377	54.1	17,721	38.7	2105	44.9	35,524	38.3	34,761	41.4	61,877	47.2	69,375	49.5	
Symptomatic	639,141	49.7	362,087	45.9	28,015	61.3	2582	55.1	57,153	61.7	49,162	58.6	69,238	52.8	70,904	50.5	
**Presence of Comorbidities**																	<0.001
No comorbidities	1,032,442	80.2	622,953	79.0	39,539	86.5	4336	92.5	67,281	72.6	65,287	77.8	109,385	83.4	123,661	88.2	
Comorbid	254,439	19.8	165,511	21.0	6197	13.5	351	7.5	25,396	27.4	18,636	22.2	21,730	16.6	16,618	11.8	

Abbreviations: AZD1222, Oxford-AstraZeneca; BNT162b2, Pfizer-BioNTech; CoronaVac, Sinovac.

**Table A2:** Fully adjusted, partially adjusted, and unadjusted vaccine effectiveness in preventing admission to ICU and deaths among COVID-19 cases, according to vaccination status with any of AZD1222, BNT162b2, or CoronaVac.

Outcomes and Vaccine Effectiveness	Vaccine Effectiveness (95% CI): Overall									
	Fully Adjusted		Unadjusted		Partially Unadjusted (No Trend and State Dummies)		Partially Adjusted (No State Dummies)		Partially Adjusted (No Comorbidities Dummy)	
	VE	95% CI	VE	95% CI	VE	95% CI	VE	95% CI	VE	95% CI
**Admission to ICU**										
Partially vaccinated	31.3	28.5, 34.1	27.2	24.5, 29.8	40.3	38.1, 42.5	28.4	25.4, 31.2	35.4	32.7, 37.9
Fully vaccinated	79.1	77.7, 80.4	74.1	72.5, 75.6	84.3	83.4, 85.3	79.0	77.6, 80.3	83.3	82.2, 84.4
**Confirmed death**										
Partially vaccinated	45.1	42.6, 47.5	15.1	12.3, 17.8	23.5	20.7, 26.2	29.9	27.2, 32.5	45.9	43.9, 47.8
Fully vaccinated	86.7	85.7, 87.6	67.5	65.7, 69.1	82.9	81.9, 83.8	85.3	84.5, 86.2	88.7	88, 89.3

Abbreviations: CI, confidence intervals; COVID-19, coronavirus disease; ICU, intensive care unit; VE, vaccine effectiveness; AZD1222, Oxford-AstraZeneca; BNT162b2, Pfizer-BioNTech; CoronaVac, Sinovac.

**Table A3:** Fully adjusted, partially adjusted, and unadjusted vaccine effectiveness in preventing admission to ICU and deaths among COVID-19 cases, according to vaccination status for AZD1222.

Outcomes and Vaccine Effectiveness	Vaccine Effectiveness (95% CI): AZD1222									
	Fully Adjusted		Unadjusted		Partially Unadjusted (No Trend and State Dummies)		Partially Adjusted (No State Dummies)		Partially Adjusted (No Comorbidities Dummy)	
	VE	95% CI	VE	95% CI	VE	95% CI	VE	95% CI	VE	95% CI
**Admission to ICU**										
Partially vaccinated	60.0	55.6, 64.0	55.6	51.0, 59.9	63.7	59.8, 67.2	55.0	50.1, 59.4	64.7	60.9, 68.2
Fully vaccinated	95.6	88.3, 98.4	95.7	88.4, 98.4	96.8	91.5, 98.8	95.4	87.8, 98.3	96.9	91.6, 98.8
**Confirmed death**										
Partially vaccinated	70.7	67.3, 73.7	34.7	29.3, 39.7	38.1	32.5, 43.3	44.8	39.6, 49.4	71.5	69.0, 73.8
Fully vaccinated	95.3	91.3, 97.4	88.2	79.2, 93.3	91.7	85.3, 95.4	93.3	88.0, 96.2	97.1	94.9, 98.4

Abbreviations: CI, confidence intervals; COVID-19, coronavirus disease; ICU, intensive care unit; VE, vaccine effectiveness; AZD1222, Oxford-AstraZeneca; BNT162b2, Pfizer-BioNTech; CoronaVac, Sinovac.

**Table A4:** Fully adjusted, partially adjusted, and unadjusted vaccine effectiveness in preventing admission to ICU and deaths among COVID-19 cases, according to vaccination status for BNT162b2.

Outcomes and Vaccine Effectiveness	Vaccine Effectiveness (95% CI): BNT162b2									
	Fully Adjusted		Unadjusted		Partially Unadjusted (No Trend and State Dummies)		Partially Adjusted (No State Dummies)		Partially Adjusted (No Comorbidities Dummy)	
	VE	95% CI	VE	95% CI	VE	95% CI	VE	95% CI	VE	95% CI
**Admission to ICU**										
Partially vaccinated	34.3	30.2, 38.1	25.2	20.8, 29.3	47.2	44.1, 50.2	36.4	32.5, 40.1	36.8	32.9, 40.4
Fully vaccinated	90.3	88.8, 91.6	88.2	86.4, 89.8	93.3	92.3, 94.2	91.2	89.9, 92.4	92.1	90.8, 93.1
**Confirmed death**										
Partially vaccinated	48.1	44.5, 51.4	14.7	10.3, 18.9	39.8	36.4, 43.1	45.1	41.9, 48.1	46.2	43.2, 49.0
Fully vaccinated	92.7	91.7, 93.6	80.9	78.8, 82.8	91.6	90.6, 92.5	92.8	92.0, 93.6	93.5	92.8, 94.2

Abbreviations: CI, confidence intervals; COVID-19, coronavirus disease; ICU, intensive care unit; VE, vaccine effectiveness; AZD1222, Oxford-AstraZeneca; BNT162b2, Pfizer-BioNTech; CoronaVac, Sinovac.

**Table A5:** Fully adjusted, partially adjusted, and unadjusted vaccine effectiveness in preventing admission to ICU and deaths among COVID-19 cases, according to vaccination status for CoronaVac.

Outcomes and Vaccine Effectiveness	Vaccine Effectiveness (95% CI): CoronaVac									
	Fully Adjusted		Unadjusted		Partially Unadjusted (No Trend and State Dummies)		Partially Adjusted (No State Dummies)		Partially Adjusted (No Comorbidities Dummy)	
	VE	95% CI	VE	95% CI	VE	95% CI	VE	95% CI	VE	95% CI
**Admission to ICU**										
Partially vaccinated	17.3	12.9, 21.4	18.3	14.3, 22.0	24.0	20.3, 27.6	10.4	5.8, 14.8	21.7	17.7, 25.6
Fully vaccinated	72.0	69.9, 73.9	64.8	62.4, 67.1	78.1	76.6, 79.5	70.3	68.1, 72.3	77.9	76.3, 79.5
**Confirmed death**										
Partially vaccinated	29.8	25.7, 33.7	8.2	4.1, 12.0	0.0	−4.9, 4.6	7.7	3.1, 12.1	31.5	28.3, 34.6
Fully vaccinated	82.4	81.0, 83.7	58.6	56.1, 60.9	75.9	74.3, 77.3	79.5	78.1, 80.8	85.6	84.6, 86.5

Abbreviations: CI, confidence intervals; COVID-19, coronavirus disease; ICU, intensive care unit; VE, vaccine effectiveness; AZD1222, Oxford-AstraZeneca; BNT162b2, Pfizer-BioNTech; CoronaVac, Sinovac.

**Table A6:** Fully adjusted, partially adjusted, and unadjusted vaccine effectiveness in preventing admission to ICU among COVID-19 cases by definitions of partial vaccination status.

Definition of Partial Vaccination Status	Vaccine Effectiveness	Cohort of Confirmed COVID-19 Cases			Vaccine Effectiveness (95% CI)									
		Total No.	Event No.	Rate of Event	Fully Adjusted		Unadjusted		No Trend and State Dummies		No State Dummies		No Comorbidities Dummy	
				(per 1000 Persons)	VE	95% CI	VE	95% CI	VE	95% CI	VE	95% CI	VE	95% CI
**1 day post dose 1**	Unvaccinated	749,524	14,497	19.3	ref	ref	ref	ref	ref	ref	ref	ref	ref	ref
	Partially vaccinated	262,441	3715	14.2	31.3	28.5, 34.1	27.2	24.5, 29.8	40.3	38.1, 42.5	28.4	25.4, 31.2	35.4	32.7, 37.9
	Fully vaccinated	227,480	1156	5.1	79.1	77.7, 80.4	74.1	72.5, 75.6	84.3	83.4, 85.3	79.0	77.6, 80.3	83.3	82.2, 84.4
	Partially vaccinated: AZD1222	45,549	395	8.7	60.0	55.6, 64.0	55.6	51.0, 59.9	63.7	59.8, 67.2	55.0	50.1, 59.4	64.7	60.9, 68.2
	Fully vaccinated: AZD1222	4677	4	0.9	95.6	88.3, 98.4	95.7	88.4, 98.4	96.8	91.5, 98.8	95.4	87.8, 98.3	96.9	91.6, 98.8
	Partially vaccinated: BNT162b2	91,793	1335	14.5	34.3	30.2, 38.1	25.2	20.8, 29.3	47.2	44.1, 50.2	36.4	32.5, 40.1	36.8	32.9, 40.4
	Fully vaccinated: BNT162b2	83,780	194	2.3	90.3	88.8, 91.6	88.2	86.4, 89.8	93.3	92.3, 94.2	91.2	89.9, 92.4	92.1	90.8, 93.1
	Partially vaccinated: CoronaVac	125,099	1985	15.9	17.3	12.9, 21.4	18.3	14.3, 22.0	24.0	20.3, 27.6	10.4	5.8, 14.8	21.7	17.7, 25.6
	Fully vaccinated: CoronaVac	139,023	958	6.9	72.0	69.9, 73.9	64.8	62.4, 67.1	78.1	76.6, 79.5	70.3	68.1, 72.3	77.9	76.3, 79.5
**14 days post dose 1**	Unvaccinated	749,524	14,497	19.3	ref	ref	ref	ref	ref	ref	ref	ref	ref	ref
	Partially vaccinated	147,032	1786	12.1	46.4	43.5, 49.2	37.7	34.5, 40.7	53.0	50.6, 55.3	43.8	40.7, 46.7	50.2	47.5, 52.8
	Fully vaccinated	227,480	1156	5.1	79.8	78.4, 81.1	74.1	72.5, 75.6	84.4	83.4, 85.3	79.7	78.3, 81.0	83.8	82.7, 84.8
	Partially vaccinated: AZD1222	35,995	248	6.9	70.0	65.9, 73.7	64.8	60.1, 69.0	72.7	69.0, 75.9	66.5	61.9, 70.5	74.2	70.7, 77.3
	Fully vaccinated: AZD1222	4677	4	0.9	95.8	88.7, 98.4	95.7	88.4, 98.4	96.8	91.6, 98.8	95.6	88.3, 98.4	97.0	91.9, 98.9
	Partially vaccinated: BNT162b2	41,916	457	10.9	55.8	51.3, 59.9	44.1	38.6, 49.1	64.6	61.1, 67.8	57.6	53.3, 61.5	57.7	53.5, 61.6
	Fully vaccinated: BNT162b2	83,780	194	2.3	90.6	89.1, 91.9	88.2	86.4, 89.8	93.3	92.3, 94.2	91.5	90.1, 92.6	92.3	91.1, 93.3
	Partially vaccinated: CoronaVac	69,121	1081	15.6	26.4	21.4, 31.2	19.4	14.3, 24.3	32.7	28.3, 36.8	20.6	15.2, 25.7	30.3	25.6, 34.7
	Fully vaccinated: CoronaVac	139,023	958	6.9	72.8	70.7, 74.7	64.8	62.4, 67.1	78.1	76.6, 79.6	71.3	69.2, 73.3	78.6	77.0, 80.0

Abbreviations: CI, confidence intervals; COVID-19, coronavirus disease; ICU, intensive care unit; VE, vaccine effectiveness; AZD1222, Oxford-AstraZeneca; BNT162b2, Pfizer-BioNTech; CoronaVac, Sinovac.

**Table A7:** Fully adjusted, partially adjusted, and unadjusted vaccine effectiveness in preventing death among COVID-19 cases by definitions of partial vaccination status.

Definition of Partial Vaccination Status	Vaccine Effectiveness	Cohort of Confirmed COVID-19 Cases			Vaccine Effectiveness (95% CI)									
		Total No.	Event No.	Rate of Event	Fully Adjusted		Unadjusted		No Trend and State Dummies		No State Dummies		No Comorbidities Dummy	
				(per 1000 persons)	VE	95% CI	VE	95% CI	VE	95% CI	VE	95% CI	VE	95% CI
**1 day post dose 1**	Unvaccinated	749,524	15976	21.3	ref	ref	ref	ref	ref	ref	ref	ref	ref	ref
	Partially vaccinated	262,441	4766	18.2	45.1	42.6, 47.5	15.1	12.3, 17.8	23.5	20.7, 26.2	29.9	27.2, 32.5	45.9	43.9, 47.8
	Fully vaccinated	227,480	1601	7	86.7	85.7, 87.6	67.5	65.7, 69.1	82.9	81.9, 83.8	85.3	84.5, 86.2	88.7	88.0, 89.3
	Partially vaccinated: AZD1222	45,549	639	14	70.7	67.3, 73.7	34.7	29.3, 39.7	38.1	32.5, 43.3	44.8	39.6, 49.4	71.5	69.0, 73.8
	Fully vaccinated: AZD1222	4677	12	2.6	95.3	91.3, 97.4	88.2	79.2, 93.3	91.7	85.3, 95.4	93.3	88.0, 96.2	97.1	94.9, 98.4
	Partially vaccinated: BNT162b2	91,793	1674	18.2	48.1	44.5, 51.4	14.7	10.3, 18.9	39.8	36.4, 43.1	45.1	41.9, 48.1	46.2	43.2, 49.0
	Fully vaccinated: BNT162b2	83,780	347	4.1	92.7	91.7, 93.6	80.9	78.8, 82.8	91.6	90.6, 92.5	92.8	92.0, 93.6	93.5	92.8, 94.2
	Partially vaccinated: CoronaVac	125,099	2453	19.6	29.8	25.7, 33.7	8.2	4.1, 12.0	0.0	−4.9, 4.6	7.7	3.1, 12.1	31.5	28.3, 34.6
	Fully vaccinated: CoronaVac	139,023	1242	8.9	82.4	81.0, 83.7	58.6	56.1, 60.9	75.9	74.3, 77.3	79.5	78.1, 80.8	85.6	84.6, 86.5
**14 days post dose 1**	Unvaccinated	749,524	15976	21.3	ref	ref	ref	ref	ref	ref	ref	ref	ref	ref
	Partially vaccinated	147,032	2687	18.3	54.9	52.3, 57.4	14.5	10.9, 18.0	32.0	28.8, 35.1	39.6	36.7, 42.4	55.4	53.3, 57.4
	Fully vaccinated	227,480	1601	7	86.9	86.0, 87.8	67.5	65.7, 69.1	82.9	81.9, 83.8	85.8	84.9, 86.6	89.0	88.3, 89.6
	Partially vaccinated: AZD1222	35,995	462	12.8	77.1	74.0, 79.9	40.3	34.5, 45.6	47.7	42.1, 52.8	54.6	49.7, 59.0	77.3	75.0, 79.5
	Fully vaccinated: AZD1222	4677	12	2.6	95.4	91.4, 97.5	88.2	79.2, 93.3	91.7	85.3, 95.4	93.5	88.5, 96.4	97.2	95, 98.4
	Partially vaccinated: BNT162b2	41,916	761	18.2	59.5	55.4, 63.2	15.1	8.6, 21.1	50.1	45.9, 53.9	55.7	51.9, 59.1	56.9	53.4, 60.2
	Fully vaccinated: BNT162b2	83,780	347	4.1	92.8	91.9, 93.7	80.9	78.8, 82.8	91.6	90.6, 92.5	93.0	92.2, 93.7	93.6	92.9, 94.3
	Partially vaccinated: CoronaVac	69,121	1464	21.2	35.8	31.0, 40.3	0.6	−4.9, 5.9	7.0	1.2, 12.4	16.6	11.3, 21.6	36.7	32.9, 40.3
	Fully vaccinated: CoronaVac	139,023	1242	8.9	82.6	81.2, 83.9	58.6	56.1, 60.9	75.9	74.3, 77.3	80.1	78.8, 81.4	85.9	84.9, 86.7

Abbreviations: CI, confidence intervals; COVID-19, coronavirus disease; ICU, intensive care unit; VE, vaccine effectiveness; AZD1222, Oxford-AstraZeneca; BNT162b2, Pfizer-BioNTech; CoronaVac, Sinovac.

## References

[1] Cascella M., Rajnik M., Aleem A., Dulebohn S.C., Di Napoli R. (2021). Features, Evaluation, and Treatment of Coronavirus (COVID-19). StatPearls [Internet].

[2] Alimohamadi Y., Sepandi M., Taghdir M., Hosamirudsari H. (2020). Determine the most common clinical symptoms in COVID-19 patients: A systematic review and meta-analysis. J. Prev. Med. Hyg..

[3] Falzone L., Gattuso G., Tsatsakis A., Spandidos D.A., Libra M. (2021). Current and innovative methods for the diagnosis of COVID 19 infection (Review). Int. J. Mol. Med..

[4] World Health Organization COVID-19 Weekly Epidemiological Update–Edition 64. https://www.who.int/publications/m/item/weekly-epidemiological-update-on-covid-19---2-november-2021.

[5] World Health Organization COVID-19 Vaccine Tracker and Landscape. https://www.who.int/publications/m/item/draft-landscape-of-covid-19-candidate-vaccines.

[6] Baraniuk C. (2021). What do we know about China’s covid-19 vaccines?. BMJ.

[7] El Sahly H.M., Baden L.R., Essink B., Doblecki-Lewis S., Martin J.M., Anderson E.J., Campbell T.B., Clark J., Jackson L.A., Fichtenbaum C.J. (2021). Efficacy of the mRNA-1273 SARS-CoV-2 Vaccine at Completion of Blinded Phase. N. Engl. J. Med..

[8] Falsey A.R., Sobieszczyk M.E., Hirsch I., Sproule S., Robb M.L., Corey L., Neuzil K.M., Hahn W., Hunt J., Mulligan M.J. (2021). Phase 3 Safety and Efficacy of AZD1222 (ChAdOx1 nCoV-19) COVID-19 Vaccine. N. Engl. J. Med..

[9] Tanriover M.D., Doğanay H.L., Akova M., Güner H.R., Azap A., Akhan S., Köse Ş., Erdinç F.Ş., Akalın E.H., Tabak Ö.F. (2021). Efficacy and safety of an inactivated whole-virion SARS-CoV-2 vaccine (CoronaVac): Interim results of a double-blind, randomised, placebo-controlled, phase 3 trial in Turkey. Lancet.

[10] Thomas S.J., Moreira E.D., Kitchin N., Absalon J., Gurtman A., Lockhart S., Perez J.L., Pérez Marc G., Polack F.P., Zerbini C. (2021). Safety and Efficacy of the BNT162b2 mRNA COVID-19 Vaccine through 6 Months. N. Engl. J. Med..

[11] Voysey M., Clemens S.A.C., Madhi S.A., Weckx L.Y., Folegatti P.M., Aley P.K., Angus B., Baillie V.L., Barnabas S.L., Bhorat Q.E. (2021). Safety and efficacy of the ChAdOx1 nCoV-19 vaccine (AZD1222) against SARS-CoV-2: An interim analysis of four randomised controlled trials in Brazil, South Africa, and the UK. Lancet.

[12] World Health Organization Considerations for Evaluation of COVID-19 Vaccines. https://www.who.int/publications/m/item/considerations-for-the-assessment-of-covid-19-vaccines-for-listing-by-who.

[13] Jara A., Undurraga E.A., González C., Paredes F., Fontecilla T., Jara G., Pizarro A., Acevedo J., Leo K., Leon F. (2021). Effectiveness of an inactivated SARS-CoV-2 vaccine in Chile. N. Engl. J. Med..

[14] Kim J.H., Marks F., Clemens J.D. (2021). Looking beyond COVID-19 vaccine phase 3 trials. Nat. Med..

[15] Bernal J.L., Andrews N., Gower C., Gallagher E., Simmons R., Thelwall S., Stowe J., Tessier E., Groves N., Dabrera G. (2021). Effectiveness of COVID-19 vaccines against the B. 1.617. 2 (delta) variant. N. Engl. J. Med..

[16] Dagan N., Barda N., Kepten E., Miron O., Perchik S., Katz M.A., Hernán M.A., Lipsitch M., Reis B., Balicer R.D. (2021). BNT162b2 mRNA COVID-19 vaccine in a nationwide mass vaccination setting. N. Engl. J. Med..

[17] Haas E.J., Angulo F.J., McLaughlin J.M., Anis E., Singer S.R., Khan F., Brooks N., Smaja M., Mircus G., Pan K. (2021). Impact and effectiveness of mRNA BNT162b2 vaccine against SARS-CoV-2 infections and COVID-19 cases, hospitalisations, and deaths following a nationwide vaccination campaign in Israel: An observational study using national surveillance data. Lancet.

[18] Vasileiou E., Simpson C.R., Shi T., Kerr S., Agrawal U., Akbari A., Bedston S., Beggs J., Bradley D., Chuter A. (2021). Interim findings from first-dose mass COVID-19 vaccination roll-out and COVID-19 hospital admissions in Scotland: A national prospective cohort study. Lancet.

[19] Krause P.R., Fleming T.R., Peto R., Longini I.M., Figueroa J.P., Sterne J.A., Cravioto A., Rees H., Higgins J.P., Boutron I. (2021). Considerations in boosting COVID-19 vaccine immune responses. Lancet.

[20] COVID-19 Vaccine Tracker Sinovac: CoronaVac. https://covid19.trackvaccines.org/vaccines/7/.

[21] World Health Organization Evidence Assessment: Sinovac/CoronaVac COVID-19 Vaccine. https://cdn.who.int/media/docs/default-source/immunization/sage/2021/april/5_sage29apr2021_critical-evidence_sinovac.pdf.

[22] COVID-19 Immunisation Task Force (CITF) Open data on Malaysia’s National COVID-19 Immunisation Programme. https://github.com/CITF-Malaysia/citf-public.

[23] (2020). Department of Statistics Malaysia. Current Population Estimates, Malaysia. https://www.dosm.gov.my.

[24] World Health Organization COVID-19 Situation Report for the Western Pacific Region #70: 8–14 September 2021. https://www.who.int/westernpacific/internal-publications-detail/covid-19-situation-report-for-the-western-pacific-region-70-8-september-2021---14-september-2021.

[25] Prime Minister Office of Malaysia Kenyataan Media Pejabat Perdana Menteri. https://www.pmo.gov.my/wp-content/uploads/2021/06/Kenyataan-Media-PMO-Pelaksanaan-Total-Lockdown.pdf.

[26] Jawatankuasa Khas Jaminan Akses Bekalan Vaksin COVID-19 (JKJAV) [The Special Committee for Ensuring Access to COVID-19 Vaccine Supply (JKJAV)]. https://www.vaksincovid.gov.my/.

[27] Ministry of Health Malaysia Open Data on the COVID-19 Epidemic in Malaysia. https://github.com/MoH-Malaysia/covid19-public/.

[28] Orenstein W.A., Bernier R.H., Dondero T.J., Hinman A.R., Marks J.S., Bart K.J., Sirotkin B. (1985). Field evaluation of vaccine efficacy. Bull. World Health Organ..

[29] Mazagatos C., Monge S., Olmedo C., Vega L., Gallego P., Martín-Merino E., Sierra M.J., Limia A., Larrauri A. (2021). Effectiveness of mRNA COVID-19 vaccines in preventing SARS-CoV-2 infections and COVID-19 hospitalisations and deaths in elderly long-term care facility residents, Spain, weeks 53 2020 to 13 2021. Eurosurveillance.

[30] Araki K., Hara M., Sakanishi Y., Shimanoe C., Nishida Y., Matsuo M., Tanaka K. (2016). Estimating rotavirus vaccine effectiveness in Japan using a screening method. Hum. Vaccines Immunother..

[31] Cohen A.L., Taylor T., Farley M.M., Schaffner W., Lesher L.J., Gershman K.A., Bennett N.M., Reingold A., Thomas A., Baumbach J. (2012). An assessment of the screening method to evaluate vaccine effectiveness: The case of 7-valent pneumococcal conjugate vaccine in the United States. PLoS ONE.

[32] Remschmidt C., Rieck T., Bödeker B., Wichmann O. (2015). Application of the screening method to monitor influenza vaccine effectiveness among the elderly in Germany. BMC Infect. Dis..

[33] Hak E., Verheij T.J., Grobbee D., Nichol K., Hoes A. (2002). Confounding by indication in non-experimental evaluation of vaccine effectiveness: The example of prevention of influenza complications. J. Epidemiol. Community Health.

[34] Van Rossum G., Drake F.L. (2009). Python 3 Reference Manual.

[35] Government of Chile Effectiveness of the SARS-CoV-2 Vaccination Program. https://www.gob.cl/en/news/sars-cov-2-vaccines-used-chile-remain-highly-effective-preventing-hospitalization-icu-admission-and-death/.

[36] Ranzani O.T., Hitchings M.D., Dorion M., D’Agostini T.L., de Paula R.C., de Paula O.F.P., de Moura Villela E.F., Torres M.S.S., de Oliveira S.B., Schulz W. (2021). Effectiveness of the CoronaVac vaccine in older adults during a gamma variant associated epidemic of covid-19 in Brazil: Test negative case-control study. BMJ.

[37] Campbell F., Archer B., Laurenson-Schafer H., Jinnai Y., Konings F., Batra N., Pavlin B., Vandemaele K., Van Kerkhove M.D., Jombart T. (2021). Increased transmissibility and global spread of SARS-CoV-2 variants of concern as at June 2021. Eurosurveillance.

[38] Planas D., Veyer D., Baidaliuk A., Staropoli I., Guivel-Benhassine F., Rajah M.M., Planchais C., Porrot F., Robillard N., Puech J. (2021). Reduced sensitivity of SARS-CoV-2 variant Delta to antibody neutralization. Nature.

[39] World Health Organization Tracking SARS-CoV-2 Variants. https://www.who.int/en/activities/tracking-SARS-CoV-2-variants/.

[40] Hashim J.H., Adman M.A., Hashim Z., Radi M.F.M., Kwan S.C. (2021). COVID-19 Epidemic in Malaysia: Epidemic Progression, Challenges, and Response. Front. Public Health.

[41] Rampal L., Liew B. (2021). Malaysia’s third COVID-19 wave-a paradigm shift required. Med. J. Malays..

[42] World Health Organization COVID-19 in Malaysia Situation Report 45. https://www.who.int/malaysia/internal-publications-detail/covid-19-in-malaysia-situation-report-45.

[43] Institute for Public Health (IPH), National Institutes of Health, Ministry of Health Malaysia (2020). National Health and Morbidity Survey (NHMS) 2019: Vol. I: NCDs–Non-Communicable Diseases: Risk Factors and other Health Problems.

[44] Centers for Disease Control and Prevention Science Brief: Evidence Used to Update the List of Underlying Medical Conditions that Increase a Person’s Risk of Severe Illness from COVID-19. https://www.cdc.gov/coronavirus/2019-ncov/science/science-briefs/underlying-evidence-table.html.

[45] National Pharmaceutical Regulatory Agency (NPRA), Ministry of Health Malaysia Frequently Asked Questions (FAQ) about COVID-19 Vaccine. https://www.npra.gov.my/.

[46] Ritchie H., Mathieu E., Rodés-Guirao L., Appel C., Giattino C., Ortiz-Ospina E., Hasell J., Macdonald B., Beltekian D., Roser M. Coronavirus Pandemic (COVID-19). https://ourworldindata.org/coronavirus.

[47] Hale T., Angrist N., Goldszmidt R., Kira B., Petherick A., Phillips T., Webster S., Cameron-Blake E., Hallas L., Majumdar S. (2021). A global panel database of pandemic policies (Oxford COVID-19 Government Response Tracker). Nat. Hum. Behav..

